# Supplementary material for: In Vitro Mucoadhesive Features of Gliadin Nanoparticles Containing Thiamine Hydrochloride
Source: Pharmaceutics. 2024 Oct 4;16(10):1296. doi: 10.3390/pharmaceutics16101296 (PMC11510220; doi:10.3390/pharmaceutics16101296)
Supplement: Supplementary file 1 [file pharmaceutics-16-01296-s001.zip › pharmaceutics-3223016-supplementary.pdf]

In vitro mucoadhesive features of gliadin nanoparticles containing thiamine hydrochloride

Silvia Voci[a], Agnese Gagliardi[a,b], Elena Giuliano[a], Maria Cristina Salvatici[c], Antonio Procopio[a,b], Donato Cosco[a,b*]

[a]Department of Health Sciences, University "Magna Græcia" of Catanzaro, Campus Universitario "S Venuta", I-88100, Catanzaro, Italy

[b]"AGreenFood" Research Center, University "Magna Græcia" of Catanzaro, Campus Universitario "S Venuta", I-88100, Catanzaro, Italy

[c]Institute of Chemistry of Organometallic Compounds (ICCOM)-Electron Microscopy Centre (Ce.M.E.), National Research Council (CNR), via Madonna del Piano n. 10, 50019 Sesto Fiorentino, Firenze, Italy

*Corresponding Author

Donato Cosco, Ph.D., Prof., Phone Number: +39 0961 369 4119, e-mail: donatocosco@unicz.it

Supplementary Information

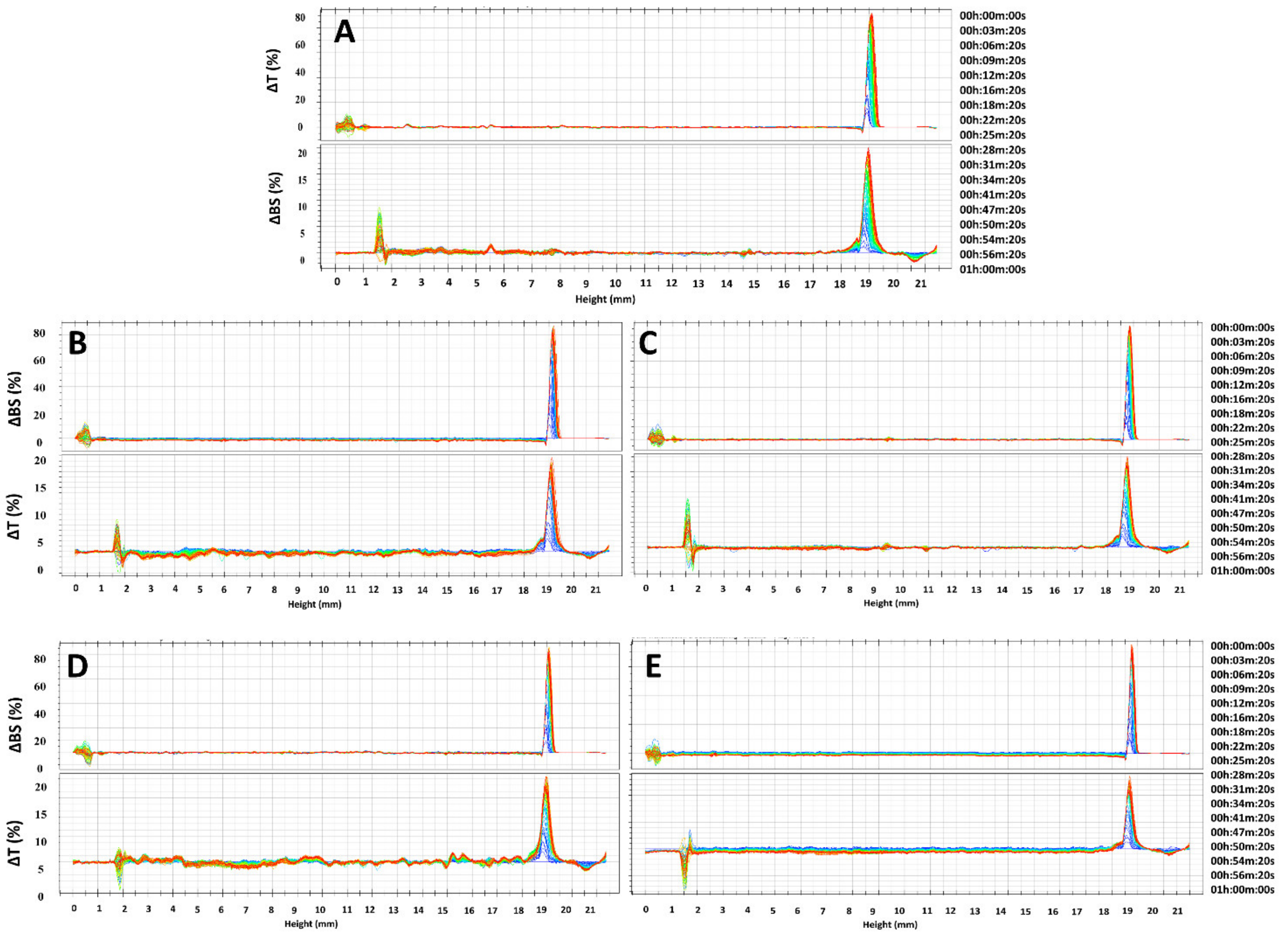

Figure S1. ΔT and ΔBS profiles of (A) empty GNPs and (B–E) B1-loaded GNPs as a function of the drug concentration and incubation time. (B) 0.2 mg/mL; (C) 0.4 mg/mL; (D) 0.6 mg/mL and (E) 0.8 mg/mL. The analysis was performed at 25 °C for one hour.

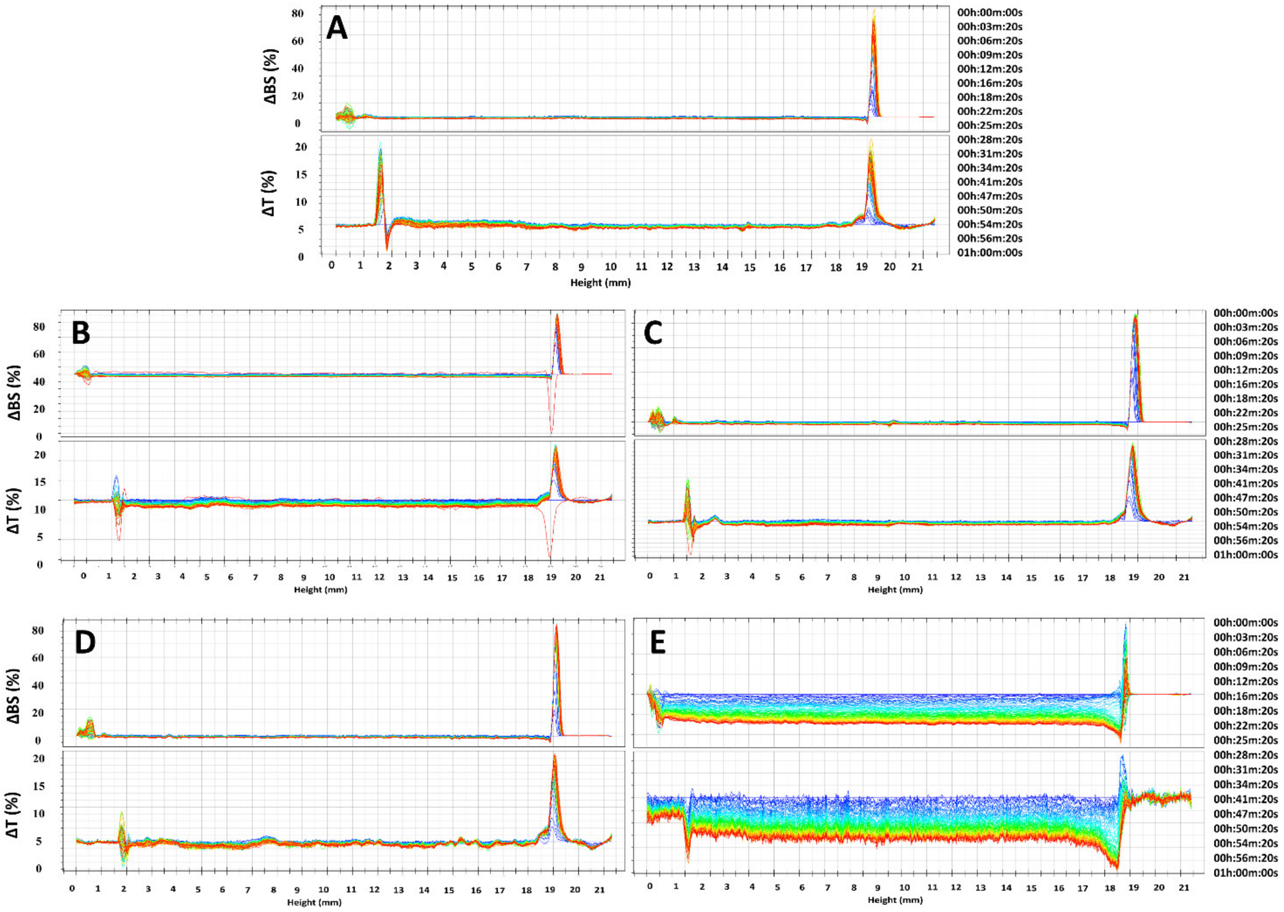

Figure S2. ΔT and ΔBS profiles of (A) empty GNPs and (B–E) B1-loaded GNPs as a function of the drug concentration and incubation time. (B) 0.2 mg/mL; (C) 0.4 mg/mL; (D) 0.6 mg/mL and (E) 0.8 mg/mL. The analysis was performed at 37 °C for one hour.

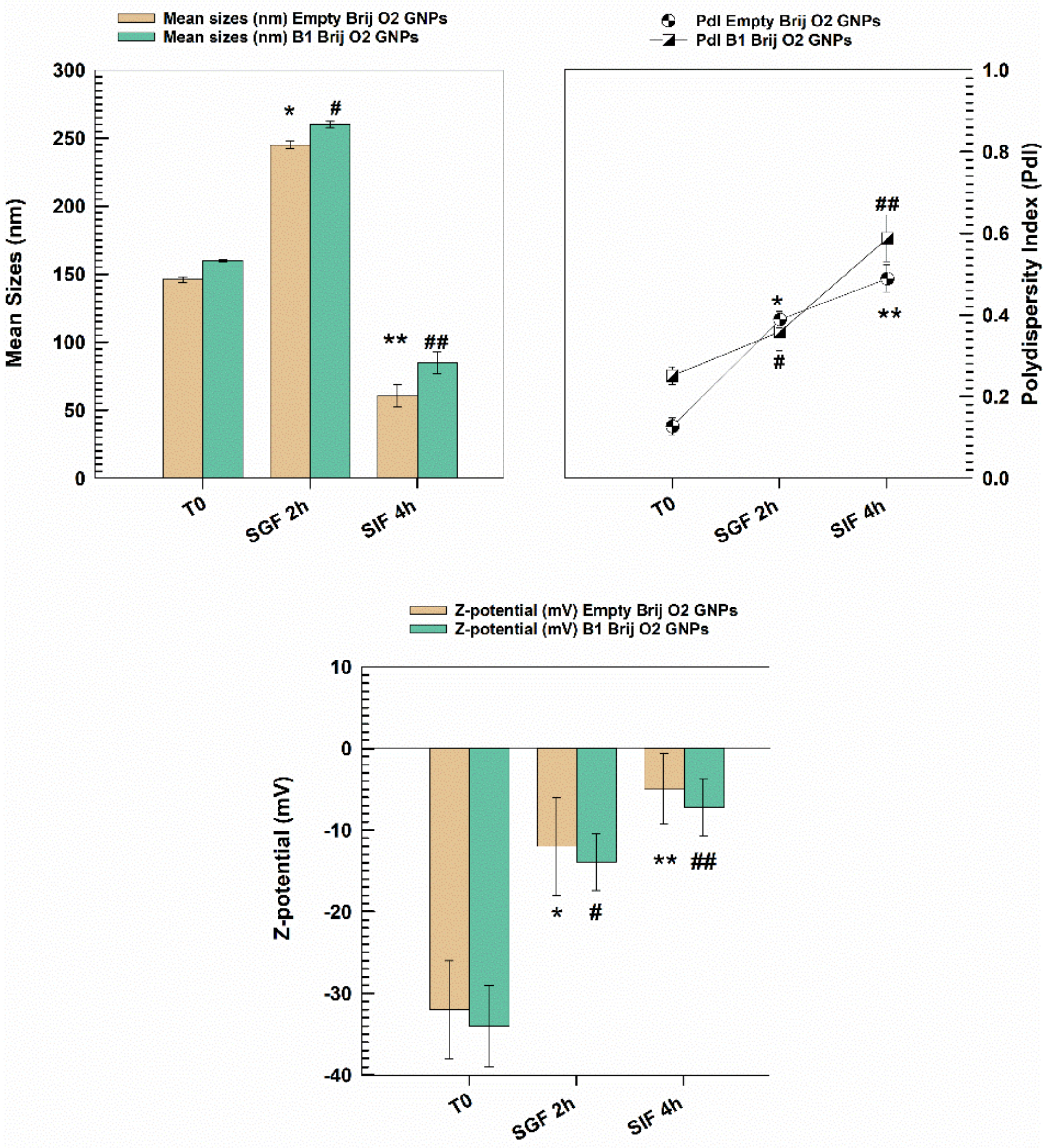

Figure S3. Mean sizes, polydispersity index and Z-potential of empty nanosystems and B1-loaded (0.6 mg/mL) Brij O2 GNPs evaluated under simulated gastrointestinal conditions. SGF (simulated gastric fluids, pH 1.2, 3.2 mg/mL of pepsin); SIF (simulated intestinal fluids, pH 7.5, 10 mg/mL of pancreatin). * and $^{\#}p<0.05$ with respect to the values obtained before incubation (T0); ** and $^{\#\#}p<0.001$ with respect to the values obtained before incubation (T0). The results are the mean of three different experiments ± standard deviation.

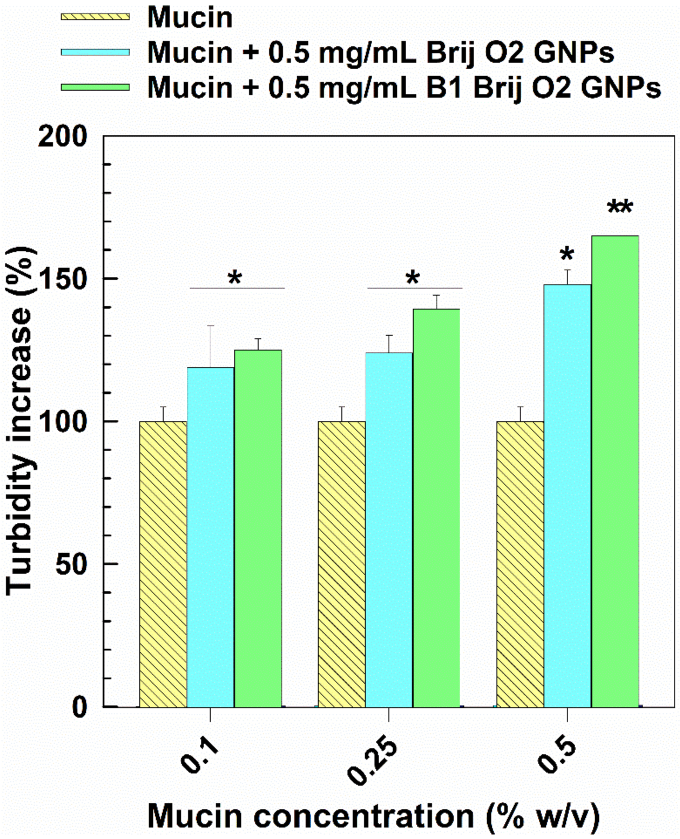

Figure S4. Turbidity increase (%) of empty and B1-loaded Brij O2 GNPs (0.6 mg/mL of B1) expressed as a function of the concentration of mucin used. The analyses were carried out at 37 °C during 4 h of incubation and are expressed as the average of three different experiments ± standard deviation. *p< 0.05 and **p< 0.001 with respect to mucin.

Table S1. TSI values of Brij O2 gliadin nanoparticles (1 mg/mL of polymer and 0.1% w/v of Brij O2) as a function of incubation time and temperature.

Sample	Drug concentration (mg/mL)	Time							
		7 (d)		14 (d)		21 (d)		28 (d)	
		Temperature							
		25 °C	37 °C	25 °C	37 °C	25 °C	37 °C	25 °C	37 °C
Brij O2 GNPs	-	4.0	4.24	4.30	4.41	4.65	4.72	4.55	4.59
B1 Brij O2 GNPs	0.2	4.29	4.63	4.40	4.13	4.22	4.50	4.62	4.69
	0.4	4.47	4.60	4.38	4.66	4.70	4.84	4.89	5.0
	0.6	4.49	4.66	4.51	4.72	4.84	4.47	4.42	4.81
	0.8	5.44	5.55	6.35	6.22	7.72	7.51	7.69	7.23

d:days.
